# Self-reported prevalence of pests in Dutch households and the use of the health belief model to explore householders’ intentions to engage in pest control

**DOI:** 10.1371/journal.pone.0190399

**Published:** 2017-12-28

**Authors:** Stefan A. Lipman, Sara A. Burt

**Affiliations:** 1 Department of Social Health & Organizational Psychology, Faculty of Social Sciences, Utrecht University, Utrecht, The Netherlands; 2 Institute for Risk Assessment Sciences, Faculty of Veterinary Medicine, Utrecht University, Utrecht, The Netherlands; University of Saskatchewan College of Agriculture and Bioresources, CANADA

## Abstract

Pests in the home are a health risk because they can be vectors for infectious disease, contribute to allergies and cause damage to buildings. The aims of this study were to record which categories of pests were reported in homes and to use a social cognition model, the health belief model, to investigate which psychological factors influence householders’ intentions to control pests. An online questionnaire was completed by 413 respondents between 11 September and 31 November 2015. A large majority of respondents reported pests in or around their home within the previous year. The prevalences were: flying insects 98%, crawling insects 85%, rodents 62%, birds 58%, and moles 20%. Regression analysis for the health belief model revealed that perceiving greater benefits and fewer barriers to pest control and expecting severe consequences of zoonotic infections predicted higher intention to control pests. Intentions towards pest control were not influenced by perceiving oneself as susceptible to catching a disease from pests or health motivation (striving towards a healthy lifestyle). Intentions to engage in pest control were lower for households reporting bird prevalence. The findings suggest that interventions aimed at improving the effectiveness of domestic pest control should focus on increasing the benefits that individuals associate with effective pest control, lowering barriers, and on underlining the severity of the diseases that pests may carry.

## Introduction

Rodents, birds and insects can be reservoirs or mechanical vectors for bacterial, parasitic and viral agents, such as *Leptospira* spp., *Salmonella* spp., *Campylobacter* spp., *Clostridium difficile*, *Chlamydia psittaci*, lymphocytic choriomeningitis virus, hantavirus and also for antibiotic resistant *Escherichia coli* [1–11]. On farms the presence of pests has been associated with antibiotic resistant strains such as methicillin-resistant *Staphylococcus aureus* and extended-spectrum beta lactamase-producing bacteria, although it is not yet clear whether the pests picked up the resistant bacteria from the farm animals or vice versa [12–15]. Non-microbial health risks associated with pests in the home are allergic asthma [16,17], the risk of chemical poisoning by inexpert biocide use [18], damage to building structures, and the danger of fire due to gnawing of electrical cables [19,20]. In view of the health risks associated with the presence of pests in the home it is desirable to encourage active pest prevention and pest control by householders and, to this end, most health authorities and local councils provide information and advice to the public.

Although several studies have investigated insects and small mammals as pests on farms [12,15,21–23], published reports on the prevalence of pests in domestic homes in the Netherlands are scarce. A study concerning the city of Amsterdam recorded rat reports in approximately 0.5–6% of homes, depending on the age of the housing stock [24], and in the same city a summary of arthropod nuisance reports registered 3,149 cases over a six year period [25]. Studies from other countries report 2–13% prevalence in domestic homes for rodents, 9–100% for flies, 12–47% for fleas, and 2–6% for cockroaches [26–30]. A number of factors influences the prevalence of pests in housing. For instance, rats are more frequently reported in and around derelict sites and housing built before 1960 than in newer buildings [24,31]. The layout of residential areas and the habits of people who live in them (e.g. the feeding of feral pigeons) can also influence pest numbers, although people can be unaware that their behaviour can encourage the presence of pests [20,32,33]. Climate change and associated migration also contribute to the spread of pest species and the diseases they carry [34].

**Fig 1:**
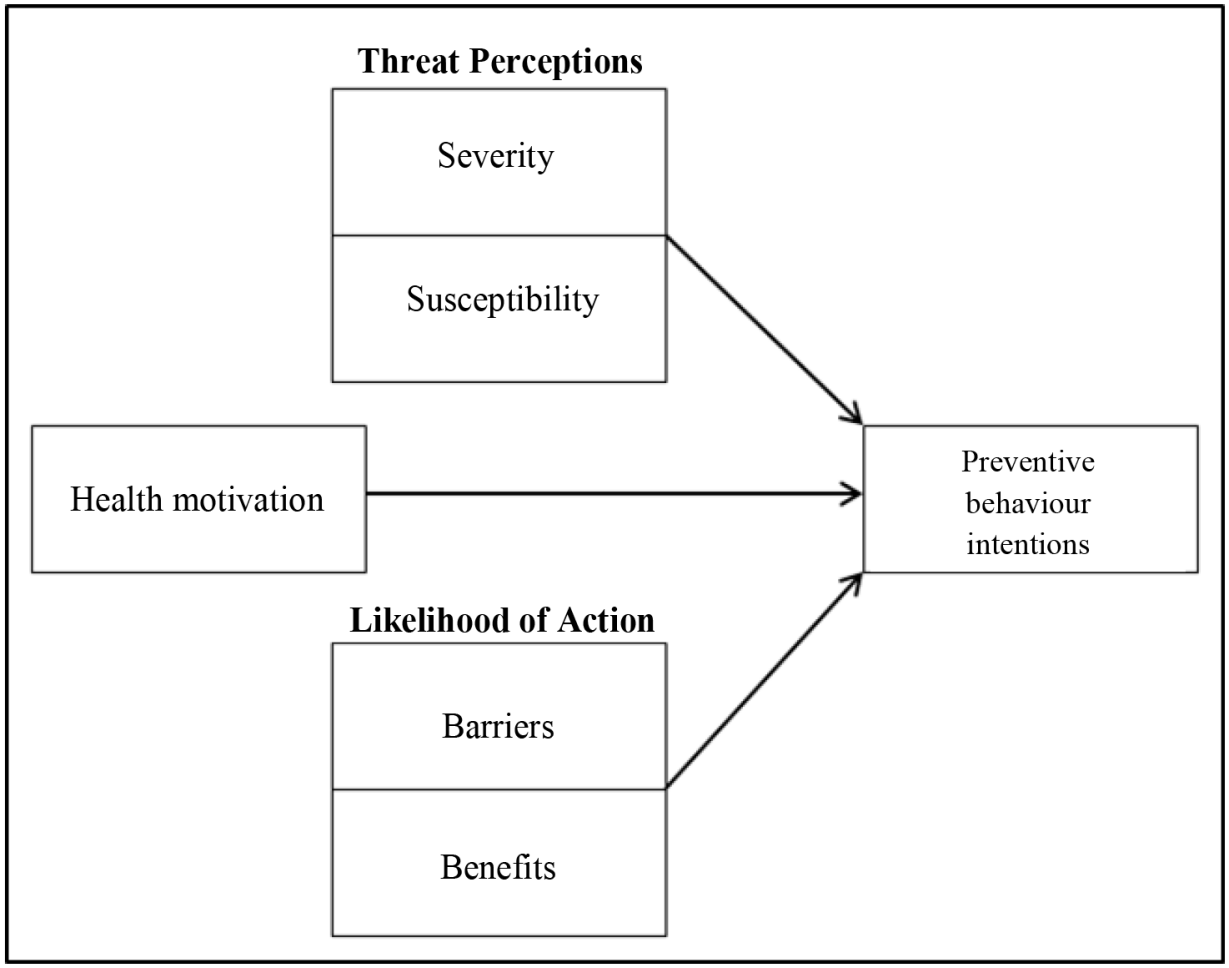
The health belief model for determinants of human behaviour (adapted from Janz & Becker 1984) [36].

Understanding the factors underlying the public’s attitude to pests and pest control would contribute to making pest control programmes more effective and to reducing the public health threat. We therefore investigated which categories of pests are reported in homes and which factors affect householders’ attitudes to pest control. A widely used social cognition model that is often used to assess attitudes in health psychology is the ‘health belief model’ (HBM) [35,36]. This model has previously been used to evaluate attitudes to various hygiene topics, such as the implementation of preventive disease control measures by cattle farmers and the use of hygienic techniques by meat handlers [37,38,39]. The HBM suggests that preventive health behaviour is determined mainly by two factors: the perceived threat to health and an evaluation of behaviour necessary to counteract this threat. The perceived threat to health comprises the perceived susceptibility to the health risk and the perceived severity of an illness; the behaviour necessary to counteract the threat comprises potential benefits and perceived barriers, such as costs (Fig 1).

The HBM is one of the most widely used social cognition models in health behaviour research [40,41]. The HBM has its origins in a study that investigated why people didn’t participate in screening for tuberculosis when it was made available [39,42] and has since been used in relation to a wide variety of health problems, including problems such as invasive species management and pest control [43].

The HBM is an intuitively logical, robust model with a finite set of well-defined constructs and is therefore relatively simple to use compared to other more complex models of health behaviour that require more data to be collected [44]. The model is useful in identifying factors in health behaviour that may influence behaviour change and fits well with symptom-prompted health behaviours [44]. It has been shown to be reliable in predicting health behaviours in both cross-sectional and intervention studies [40].

One of the limitations of the HBM is that it is based on cognition and does not have an explicit separate component for emotions [40]. Another limitation is that there is discussion over the number and inter-relatedness of constructs in the model. Interactions between the constructs and their weighting relative to each other have not yet been fully investigated [40,41]. There have also been proposals to add more constructs to the model. For example, Rosenstock et al. proposed that self-efficacy, a belief in one’s own capability to successfully execute the necessary course of action, should be added to the HBM as a separate construct [45]. However, a perceived lack of self-efficacy could be classed as one of the barriers to taking action and, as such, one could consider that this factor is already included in the model [46]. On balance, it is possible that self-efficacy influences health behaviour both directly (as a separate construct) and indirectly (via perceived barriers) [36,40]. In the present study concerning pest control in private dwellings, householders would have a choice between carrying out pest control themselves or engaging a pest control contractor. This dual interpretation of self-efficacy in this study would complicate the construct and detract from the clarity of the model. In view of this, and because it has not yet been confirmed that adding self-efficacy to the HBM increases the validity of the model [40], we chose to exclude self-efficacy as a separate component in the present study.

Some versions of the HBM include the construct ‘cues to action’ [36,39,42]. In the context of pest control an example of a cue to action would be seeing a mouse. However, cues have also been suggested to operate chiefly through the construct of the perceived threat [47] and, since this study investigates the *intention* to control pests and was not carried out in an infestation situation, cues were not included as a separate construct in the model. The version of the HBM selected for this study (Fig 1) has been used in earlier studies in related fields of hygiene research [37].

The study hypothesis was that householders’ intentions to engage in pest control can be predicted using the HBM. The objectives of this study were (i) to record the reported prevalence of pests in households and (ii) to use the health belief model to investigate factors that influence householders’ attitudes towards pest control.

## Materials and methods

### Study population & study design

A Dutch language online questionnaire was used for the study, so all Dutch speakers with access to the internet were eligible to participate. The study used a correlational design.

### Sampling method

A convenience sampling method was used to distribute an online questionnaire powered by Google Forms software and made available through Utrecht University’s website from 11 September to 30 November 2015. The aim was to obtain as large a sample of citizens as possible living in both rural and urban areas. To encourage participation from as broad a public as possible, links to the survey were placed on the websites of the health authority (GGD Regio Utrecht) and the Dutch home owners’ association (Vereniging Eigen Huis) and placed on Twitter and Facebook.

Participants contributed data anonymously and were notified at the beginning of the survey that their data would be used anonymously and only for the present study. As this is an observational study in which personal experiences and opinions are not manipulated, consultation with an Ethics or Data Protection Committee was not required. The study complies with the Dutch Data Protection Act. The STROBE checklist of items that should be included in reports of cross-sectional studies is included in S3 File.

### Questionnaire design

After clicking on the web link, participants were directed to the first page where the purpose of our study was explained. The subject was introduced, and participants could read that the study was aimed at investigating the prevalence of pests in households. Anonymity was ensured because no identifying personal data were recorded. In total the survey was expected to take 10–15 minutes to fill in. To ensure that participants knew which species should be viewed as pests the following definition was provided: ‘Many may hold different views of what pests are. To remove uncertainty, we provide a list of what species are considered to be pests for the present purpose: 1) rodents (e.g. mice, rats), 2) flying insects (e.g. mosquitos, flies, wasps), 3) crawling insets (e.g. cockroaches, fleas, silverfish), 4) birds (e.g. pigeons, crows), 5) moles.’ Participants filled in a demographic questionnaire. Then they were presented with several statements that measured the constructs of the HBM and were asked to indicate if they had encountered the five pest categories listed in or around their home during the previous year. Prior to the online launch, the survey was tested by inviting four inhabitants of the local town and two members of the university technical staff to complete a pilot questionnaire, after which the questionnaire was adjusted. The adjustments required were as follows: removing ambiguous wording and placing the responses to questions in the same order for all questions. No major adjustments were necessary.

#### Demographics questionnaire

This recorded: gender, age, education level, house type, age of house (i.e. built before or after 1970), area of residence (rural or urban). Because the survey was launched via a university website, we included a question on whether participants were full-time students and, as a follow-up, whether they lived with their parents or by themselves. Participants were also asked whether they owned pets or farm animals.

#### HBM-questionnaire

This contained 28 statements single-sentence statements and participants were asked to indicate to what extent they agreed with the statements on a 5-point Likert scale (1–5, from Totally disagree—Totally agree). Five HBM-constructs were used: threat susceptibility, threat severity, perceived benefits, perceived barriers and health motivation. The statements were constructed by selectively adapting statements to fit pest control as an outcome from parts of questionnaires from earlier studies (Champion, 1984 [48] and Nexøe, Kragstrup & Sogaard, 1998 [49]). This questionnaire was not validated beyond the prior test described earlier, and serves as a first test of the relevance of HBM for pest control. Each of the HBM constructs was measured using five statements. As a measure of preventive behaviour, the intention to engage in pest control was measured using three statements.

#### Pest prevalence questionnaire

This listed the five pest categories and participants were asked to indicate how often they had encountered these pest categories in and around their home in the past year. They noted their answers in the appropriate box: Never—Once—A few times—Regularly—Often.

The original questionnaire and an English translation are provided in S1 and S2 Files.

### Data analysis

The data was analysed using SPSS version 21 (IBM Corp., Armonk, NY). Descriptive statistics and frequencies were obtained for all complete responses and pairwise exclusion was used to account for missing data. The HBM constructs were determined by performing reliability analyses, and subsequently calculating the mean of each scale. To test the hypothesis that intentions to engage in pest control can be predicted using the HBM, we used multiple regression analyses with HBM constructs as independent variables. Additionally, we tested whether predictors should be added above the HBM constructs by running univariate tests for each demographic variable and adding predictors which significantly affect intentions to engage in pest control to the model. All tests used a significance level of alpha = 0.05.

## Results

### Demographic characteristics of survey participants

A total of 413 people completed the online survey and the demographic characteristics are presented in Table 1. Two thirds of the respondents were female. Ages were fairly evenly distributed over the three categories of young adults, middle aged and above 50. Although recruitment occurred through university channels, only 25% of the sample were students. Approximately 60% of the respondents had achieved a university education. Most participants lived either in terraced houses or apartments and a large majority lived in urban areas. More than half reported being animal-owners. These data suggest that the survey participants reflect a relatively wide array of the Dutch population. Of the 413 respondents, 411 submitted data on the prevalence of pest animals in and around the home during the past year.

**Table 1:** Demographic characteristics of survey participants.

Demographic variables	Frequency (n = 413)	Percent (%)
**Age group (Years)**		
17–25	105	26.1
26–50	200	49.6
51+	98	24.3
**Gender**		
Male	126	30.5
Female	284	68.8
**Education level**		
Primary school	0	0.0
High school	54	13.3
College education	81	19.9
University education	246	60.4
**Full-time student**	104	25.8
**House built**		
Before 1970	150	36.3
After 1970	237	57.4
Unknown	23	5.6
**House type**		
Terraced house	163	39.5
Flat/apartment	115	27.8
Semi-detached house	19	4.6
Farm	51	12.3
Other	55	13.3
**Pet/animal owners**	220	54.1
**Area of Residence**		
Rural	122	29.5
Urban	288	69.7

### Reported prevalence of pests in homes during the past year

**Fig 2:**
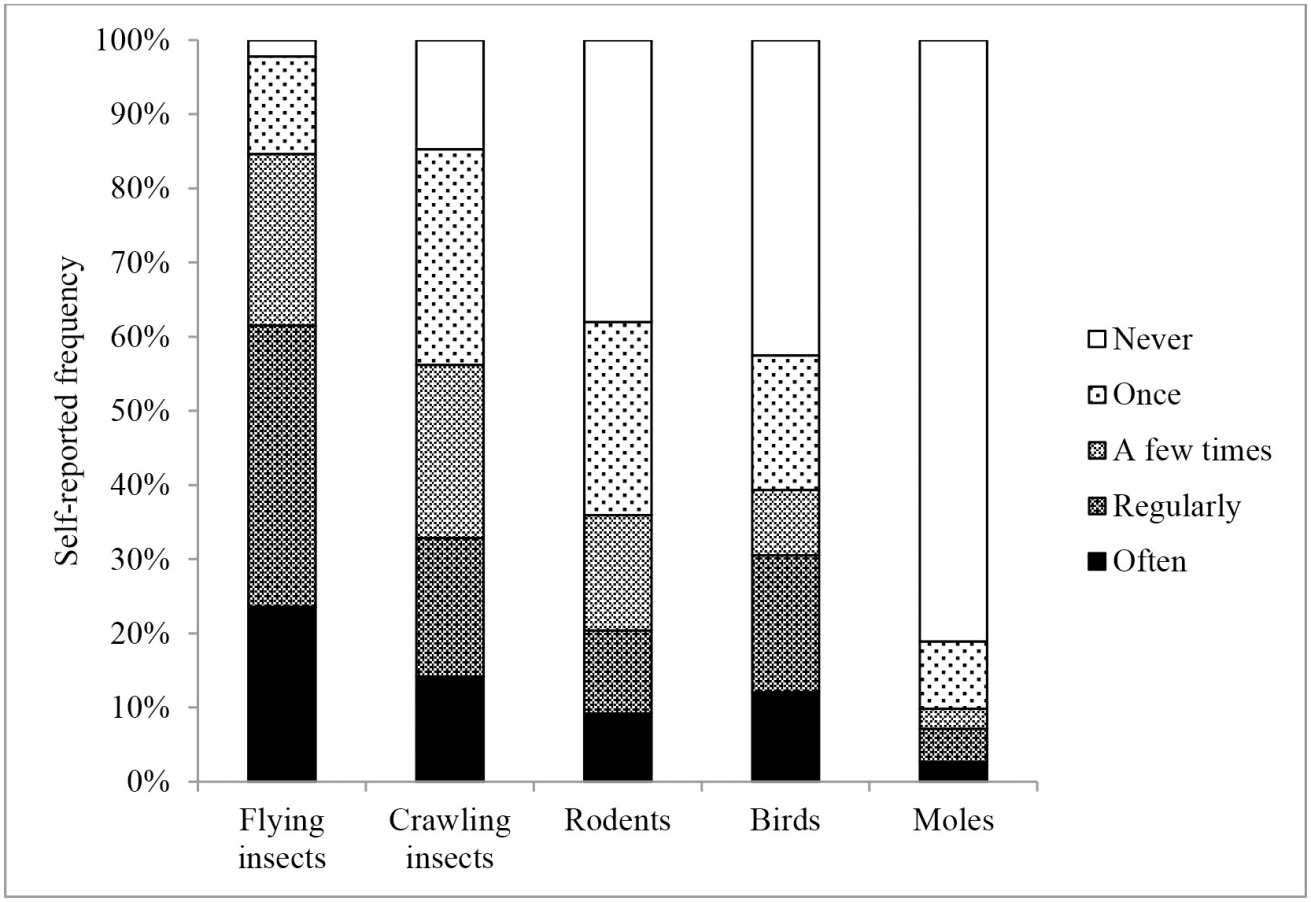
Reported frequency of pest sightings in homes during the past year (n = 411).

Respondents were asked to indicate which of the pest categories they had seen in or around the home during the past year: rodents, flies, crawling insects, birds and moles and the frequency with which these pests had been sighted. The results of the self-reported prevalence of pests from 411 respondents are presented in Fig 2 and in S1 Table. Only two percent of respondents reported no pests in the home during the past year. Flying insects were most frequently reported (98% of respondents), followed by crawling insects (85%), rodents (62%) and birds (58%). Moles were reported the least often: 20% overall. Moles, however, live in grass and lawns and therefore cannot be a pest of individual flats or apartments. After exclusion data related to living in a flat/apartment, moles were reported by 25% of respondents. Rats were reported by 119/150 (79%) of respondents who lived in houses built before 1970 and by 119/237 (50%) of respondents who lived in houses built after 1970.

### HBM scale construction

To determine the HBM scales, internal consistency was first determined using Cronbach’s α. Table 2 shows the results reliability of these scales, and the means for the scales after recoding and excluding two questions to improve internal consistency (see S2 Table for excluded items). Out of the six scales constructed to be used in further analyses, two scales have lower internal consistency than the cut-off score of 0.7 that is typically regarded as desirable [50,51]. Although reliability was sub-optimal for these scales, in view of recent discussions on the limited value of cut-off scores for Cronbach’s α [52] we included these scales in this first attempt to explore householders intentions using the HBM.

**Table 2:** The means, standard deviations (SD) and internal consistency (Cronbach’s α) of the combined HBM scales.

Scale	Mean	SD	α
Health motivation	4.07	0.66	0.72
Perceived benefits ^a^	3.78	0.64	0.71
Perceived barriers ^a^	2.97	0.78	0.61
Threat severity	2.96	0.74	0.62
Threat susceptibility	1.50	0.64	0.85
Intention to engage in PC	4.00	0.82	0.82

^a^. Cronbach’s α after removing one item from the scale

### Regression analyses

To test whether intentions to engage in pest control could be predicted using the HBM, multiple regression analyses were performed with intentions to pest control as the dependent variable (see Table 3 for regression coefficients). To test whether any predictors above the theoretically relevant HBM variables should be added to the regression model, first simple correlations (for ordinal/ratio data) and t-tests or ANOVA’s (for nominal data) were calculated with intentions to engage in pest control as dependent variable. We also included the reported pest prevalence in this first step, by coding dummy variables for each pest. For example, bird-type would be a dummy variable distinguishing between participants who would have seen no birds in or around their house, or those who reported having seen birds at least once. Through this procedure we are able to obtain a theoretically relevant, yet parsimonious model, with high statistical power. No significant correlations were observed between intention to engage in pest control and age (*r* = -.02, *p* = .68), and education level (*r* = -.03, *p =* .60). We found no differences in intentions to engage in pest control for the following demographics: gender, currently studying, pet ownership, area of residence, house build year (all *t’s <* 1.79, *p’s >* .08*)*. When comparing intentions to engage in pest control for pest-type dummies, we find no differences for all pest types (all *t’s* < 1.94, *p’s* >.06), with the exception of birds (t (400) = 2.22, *p* = .03), where participants reporting birds have lower intentions to engage in pest control (M = 3.92, SD = .86) than those who did not encounter birds (M = 4.10, SD = .76). For our analyses, it makes no difference whether pest-type is categorized as a dummy variable or treated as interval type variable (with a continuous scale). In both cases, only the reporting of birds remains of significance. Therefore we ran our model twice, firstly only including theoretically relevant HBM concepts, and secondly including the degree of bird-reporting in the model.

**Table 3:** Regression coefficients for multiple regression analysis of HBM constructs, with spearman and semi-partial correlation coefficients.

*Predictors*	*B*	*SE B*	*β*	*95% CI (B)*	*R2*	*Adj*. *R2*	*P (F change)*
**Model 1**					.0.21	0.20	<.001
					Simple correlation		Semi-partial correlation
Health Motivation	.01	.06	.007	(-.10 / .12)	.03		.01
Perceived barriers	-.19	.05	-.18 ***	(-.29 / -.09)	-.11 *		-.17
Perceived benefits	.63	.07	.39 ***	(.49 / .78)	.40 ***		.38
Threat Severity	.20	.05	.18 ***	(.09 / .30)	-.17 **		.17
Threat Susceptibility	.07	.07	.05	(-.06 / .20)	.09		.05
					***R2***	***Adj*. *R2***	***P (F change)***
**Model 2**					0.24	0.22	0.001
					Simple correlation		Semi-partial correlation
Health Motivation	.03	.06	.02	(-.11 / .11)	-		.02
Perceived barriers	-.18	.05	-.17 ***	(-.28 / -.08)	-		-.16
Perceived benefits	.62	.07	.38 ***	(.48 / .76)	-		.38
Threat Severity	.19	.05	.18 ***	(.09 / .30)	-		.16
Threat Susceptibility	.08	.06	.06	(-.05 / .21)	-		.06
Bird-type	-.08	0.03	-.15**	(-.13 / -0.04)	-.17***		-.15

Note:

*, **, and *** signify significance at *p* < .05, *p* <.01 and *p* <.001 respectively

As shown in Table 3, Model 1 significantly predicts intentions to engage in pest control (R^2^ = 0.21, ΔF (5, 396) = 21.45, p <.001). Of the HBM constructs, only perceived benefits (β = 0.39, t (396) = 8.59, p <0.001, 95% CI [.49, .78]), perceived barriers (β = -0.19, t (396) = -3.79, p <0.001, 95% CI [-.29, -.09]), and threat severity (β = 0.20, t (396) = 3.71, p < 0.001, 95% CI [.09, .39]) significantly predicted the intentions to engage in pest-control. Inclusion of bird-type pest reporting (Model 2) significantly improved the model (R^2^ = 0.24, ΔF (1, 395) = 11.73, p = .001), with bird reporting significantly predicting intentions to engage in pest control (β = -0.08, t (395) = -3.43, p = 0.001, 95% CI [-.13, .04]). No profound differences are observed for the HBM constructs between Model 1 and Model 2. These results indicate that perceiving more benefits and fewer barriers to pest control, in addition to viewing infectious diseases as more serious predicts higher intentions to engage in pest control. These results also show that intentions to engage in pest control are not dependent on how susceptible to disease respondents view themselves or their motivation to live a healthy lifestyle. Higher reporting of birds in and around the house is associated with lower intentions to engage in pest control.

## Discussion

The study objectives were to record the reported prevalence of pests in participating households and to use the HBM to investigate factors that influence householders’ attitudes towards pest control. This study is the first to assess reported numbers of all categories of pests in Dutch homes. This is also the first known study to apply the HBM to the topic of pest control and to identify some of the factors that influence householders’ intentions to engage in pest control.

### Self-reported prevalence of pests in and around the home

Insects were most frequently reported, followed by rodents, birds and then moles. The fact that insects were reported by the highest proportion of respondents (98%) and sighted most frequently over the preceding year compared to other pest categories (Fig 2) coincides with the higher end of the range reported by studies in the USA (9–100%) [26,27]. Crawling insects were reported by 85% of respondents in the present study (Fig 2). This aligns with data from an inventory of all insects in 50 homes by entomologists in the USA, which revealed that 100% of homes contained beetles and 10% fleas [27]. Crustaceans (e.g. woodlice), which can resemble crawling insects to an inexpert eye, were reported in 86% of the US sample [27].

The percentage of respondents reporting rodents in or around their home during the past year (62%) (Fig 2) is much higher than reported data for the UK and USA (2–13%) [26,29,30,53] and tenfold higher than recent reports for the city of Amsterdam (up to 6% in housing built <1960) [24]. However, recordings of up to 50% of properties with mouse infestations have been recorded in inner city ‘hot spots’ [54]. Our data will probably be influenced by selection bias, since people who have recently experienced rodent problems may be more likely to take part in the survey than those who have not [55]. Interestingly, respondents living in older houses (built before 1970) reported rats more frequently than respondents living in newer houses (built after 1970) (79% as opposed to 50%). This trend agrees with the findings of Van Adrichem et al. [24].

Birds were reported as a pest by the majority of respondents (58% prevalence) (Fig 2). This appears to be the first published report of birds being perceived as a pest around homes in The Netherlands. In contrast, some bird species have long been recognised as pests in public areas and airports and many urban councils have policies regarding feral pigeon control [6,7,32,56]. Winter feeding of garden birds contributes to the numbers of birds seen around the home and is a pastime enjoyed by the majority of householders, according to a UK study [57]. This may partly explain our finding that respondents reporting birds as pests have a lower intention to pest control. It is possible that householders who feed wild birds can find them a nuisance when in large numbers or when the feeding activities attract larger species more often seen as pests (such as crows or gulls), but, because these people enjoy engaging with birds, they are reluctant to take action to control their numbers.

Moles were the least reported of all pest categories: 20% of all respondents (Fig 2). In earlier published reports of moles as household pests no prevalences were recorded [58,59].

### The use of the HBM to predict intentions towards pest control

Our findings indicate that the most important factors affecting pest control intentions are: the benefits and barriers perceived, and the expected severity of potential disease. This indicates that it may be unimportant whether individuals view themselves as susceptible to diseases carried by pests. Apparently, caring about one’s health, even to a great extent, does not imply an intention to control pests if they are detected in the home. Our finding that susceptibility is a weak predictor agrees with a meta-analysis of the HBM [60]. Our finding that benefits, barriers and severity are predictive concurs with a review of the relative size of HBM constructs in health care studies. In that paper, however, perceived barriers carried most weight, followed by benefits and susceptibility and then severity [40].

### Implications of this study

The self-reported prevalence of pests in homes presented here suggests an extensive potential health hazard in homes. To obtain a more precise and objective inventory of the size of the pest problem, a study including objective recording of pests by experts in randomly selected homes would be warranted.

The findings of the HBM analysis could be translated into policy fairly easily. Intervention aimed at motivating householders to engage in pest control could focus on (i) emphasising the benefits and barriers that individuals associate with effective pest control, and (ii) underlining the severity of the diseases that pests may carry. Attention should also be paid to the fact that when birds are reported as pests, individuals may be reluctant to take measures to control the numbers.

The use of rodenticides is becoming increasingly restricted due to genetic resistance to chemical rodenticides [61,62] and changes in regulations that aim to reduce secondary poisoning of non-target wildlife species [63]. Sustainable methods of pest prevention and control based on integrated pest management (IPM) are therefore increasingly needed [64]. A better understanding of the beliefs, motivations and attitudes of the public towards pests and their control will enable more effective preventive pest control and, thereby, further reduction of associated health risks.

### Limitations of this study

The pest prevalences are self-reported and not objectively measured data; inaccuracies could stem from faults in reporting. Social stigma attached to the presence of pests in the home may suppress self-reported prevalences [19]. However, since the online survey was anonymous, the authors do not expect that this stigma would apply to this study. On the other hand, selection bias may have artificially raised prevalences: since people who have recently experienced a pest problem may be more likely to take part in such a survey than people who have not [55]. Since the study was carried out via an online survey, only people who use internet would be able to participate. This may have caused selection bias, for example, to a younger average age in the sample group. A survey carried out by experts in randomly chosen homes would provide more objective data.

A limitation to this HBM analysis is the fact that only correlational data was generated since the HBM constructs (perceived threat to health, perceived severity, potential benefits and perceived barriers) were all measured at the same time point and no experimental manipulations were performed. Also, this study measured *intentions* to engage in pest control. Behavioural studies have demonstrated that a discrepancy exists between intentions and actual behaviour, i.e. the ‘intention behaviour gap‘[65]. To tackle both these points, a prospective follow-on study could be planned to determine whether increasing perceived benefits would lead to a greater willingness to engage in pest control.

Some scales derived from the questionnaire that was used to measure HBM constructs had low to acceptable internal consistency i.e. correlations between the questions aimed at measuring the same construct. This indicates that the questions used in our instrument are not necessarily measuring the same variables. From a methodological point of view, this may have been caused by the fact that we aimed to capture evaluations of all categories of pests in each question. Greater reliability may have been achieved through focusing the study on one particular pest category. From a statistical point of view, the lower scores for Cronbach’s α can be explained as the result of the questionnaire design, which consisted of relatively few questions and answering options, both of which are known to produce low α scores [66].

In cross-sectional studies such as this, it is possible that participants’ previous experience may influence perceptions for the future [67]. For example, if one has had to contend with pests in the home in the past, that experience will influence the response to questions on the perceptions, particularly susceptibility. In contrast, if one has never had pests in the home, this source of bias is absent. A psychological model that incorporates the HBM with other theories may be better suited to this topic. The recently developed ‘behaviour change wheel’ whose main constructs are capability, opportunity and motivation [68], could be useful to improving pest control activities, as proposed by McLeod et al [43].

### Suggestions for further research

An objective survey by specialists of the numbers and species of pests present in a number of randomly selected homes would provide a more accurate picture of pest prevalence. To investigate whether the changes in local government communications to the public on pest control proposed here do indeed influence intentions to engage in pest control, a prospective study could be carried out.

## Conclusions

This study shows that ninety eight percent of respondents report pests in or around the home during the past year. The frequency of sightings decreases in the order: flying insects, crawling insects, rodents, birds, moles. Householders’ intention to engage in pest control can be described by factors described in the HBM for human health behaviours. The intention to carry out pest control is dependent on the perceived benefits, perceived barriers and threat severity but not on perceived susceptibility to zoonoses or health motivation. Individuals who report birds as pests are less likely to engage in pest control than others. These findings could be used to develop the central message of local government and health authority communications on pest control so as to engage the public better and to make pest control activities more effective.

## Supporting information

S1 File Questionnaire on household pests and intentions to engage in pest control (original, in Dutch). (PDF)

S2 File Questionnaire on household pests and intentions to engage in pest control (English translation). (PDF)

S3 File STROBE checklist. Checklist of items that should be included in reports of cross-sectional studies.
(PDF)

S1 Table Survey responses regarding pest prevalence. (PDF)

S2 Table Response frequencies to questions relating to the HBM. (PDF)
