# Supplementary material for: Self-reported prevalence of pests in Dutch households and the use of the health belief model to explore householders’ intentions to engage in pest control
Source: PLoS One. 2017 Dec 28;12(12):e0190399. doi: 10.1371/journal.pone.0190399 (PMC5746277; doi:10.1371/journal.pone.0190399)
Supplement: S1 File — (PDF) [file pone.0190399.s001.pdf]

# Plaagdieren-enquête 2015

Geachte deelnemer,

Veel mensen komen in aanraking met plaagdieren in of om het huis. Toch is er maar weinig bekend over het aantal keren dat dit voorkomt, en hoe mensen daarmee om gaan. Daarom doet de Universiteit Utrecht onderzoek naar de mate waarin plaagdieren voorkomen in de Nederlandse huishoudens.
Deze korte vragenlijst dient als een eerste meting. Graag zouden we u een aantal vragen willen stellen, het invullen van deze vragen neemt 5 tot 10 minuten in beslag. Uw antwoorden blijven GEHEEL ANONIEM, en worden alleen voor wetenschappelijke doeleinden gebruikt.

Niet iedereen zal onder plaagdieren hetzelfde verstaan. Om onduidelijkheid te voorkomen geven we u een lijst van dier- en insectsoorten die voor dit doel als plaagdieren kunnen worden beschouwd:
- knaagdieren (bijvoorbeeld: muizen, ratten, e.d.)
- vliegende insecten (bijvoorbeeld: muggen, vliegen, wespen, e.d.)
- kruipende insecten (bijvoorbeeld: kakkerlakken, vlooien, zilvervisjes, e.d.)
- vogels (bijvoorbeeld: duiven, kraaiachtigen, e.d)
- mollen

Bij voorbaat dank voor uw deelname.

## Algemene gegevens

1. **Opleiding**
   Volgt u op dit moment een voltijdsopleiding?
   *Markeer slechts één ovaal.*
   - ( ) Ja
   - ( ) Nee

2. **Woongebied**
   Waar is uw woning gevestigd?
   *Markeer slechts één ovaal.*
   - ( ) Dorp
   - ( ) Stad
   - ( ) Platteland

3. **Leeftijd**
   Wat is uw leeftijd?

   ______________________________

4. **Opleidingsniveau**
   Wat is uw hoogst afgeronde opleiding?
   *Markeer slechts één ovaal.*
   - ( ) Basisschool
   - ( ) vmbo
   - ( ) havo
   - ( ) vwo

- mbo
- hbo
- universitair
- Anders: ______________________

5. **Sekse**

Wat is uw geslacht?
*Markeer slechts één ovaal.*

- Man
- Vrouw

6. **Huisdieren**

Heeft u dieren in of om uw woning?
*Markeer slechts één ovaal.*

- Nee
- Ja, ik heb een of meerdere huisdieren
- Ja, ik houd beroepsmatig dieren in of om mijn woning
- Ik heb zowel een of meerdere huisdieren als beroepsmatig vee

7. **(alleen voor studenten) Uitwonend**

Bent u een uitwonende student?
*Markeer slechts één ovaal.*

- Ja
- Nee

8. **Bouwjaar**

Wanneer is uw woning (ongeveer) gebouwd?
*Markeer slechts één ovaal.*

- Voor 1960
- Na 1960
- Niet bekend

9. **Type huis**

In wat voor soort woning woont u?
*Markeer slechts één ovaal.*

- Rijtjeshuis / hoekhuis
- Flat of bovenwoning
- Benedenwoning
- Twee onder 1 kap
- Veehouderij
- Vrijstaand huis
- Overig

## Stellingen

Hieronder vind u een aantal stellingen die betrekking hebben op uw gezondheid, plaagdieren en plaagdierbestrijding. U kunt telkens aangeven in hoeverre u het eens bent met deze stelling, door een van de antwoordopties aan te vinken. De antwoordopties zijn voor elke stelling hetzelfde, die zijn namelijk:

A) Helemaal mee oneens
B) Deels mee oneens
C) Neutraal
D) Deels mee eens
E) Helemaal mee eens

## Gezondheid

10. **Ik volg de voorschriften van mijn huisarts omdat ze mijn gezondheid bevorderen**
*Markeer slechts één ovaal.*

- ( ) Helemaal mee oneens
- ( ) Deels mee oneens
- ( ) Geen mening
- ( ) Deels mee eens
- ( ) Helemaal mee eens

11. **Ik heb een gebalanceerd eetpatroon**
*Markeer slechts één ovaal.*

- ( ) Helemaal mee oneens
- ( ) Deels mee oneens
- ( ) Geen mening
- ( ) Deels mee eens
- ( ) Helemaal mee eens

12. **Mijn gezondheid is belangrijk voor me**
*Markeer slechts één ovaal.*

- ( ) Helemaal mee oneens
- ( ) Deels mee oneens
- ( ) Geen mening
- ( ) Deels mee eens
- ( ) Helemaal mee eens

13. **Ik sport regelmatig**
*Markeer slechts één ovaal.*

- ( ) Helemaal mee oneens
- ( ) Deels mee oneens
- ( ) Geen mening

- Deels mee eens
- Helemaal mee eens

14. **Ik doe vaak dingen om mijn gezondheid te verbeteren**

(e.g. sporten, vroeg naar bed gaan, gezond eten)

*Markeer slechts één ovaal.*

- Helemaal mee oneens
- Deels mee oneens
- Geen mening
- Deels mee eens
- Helemaal mee eens

15. **Ziektes opgelopen via plaagdieren zijn ernstig**

*Markeer slechts één ovaal.*

- Helemaal mee oneens
- Deels mee oneens
- Geen mening
- Deels mee eens
- Helemaal mee eens

16. **Ik schat de kans dat ik ziek word door plaagdieren in en om mijn woning hoog in.**

*Markeer slechts één ovaal.*

- Helemaal mee oneens
- Deels mee oneens
- Geen mening
- Deels mee eens
- Helemaal mee eens

17. **Ik verwacht binnen het jaar ziek te worden door plaagdieren in en om mijn woning**

*Markeer slechts één ovaal.*

- Helemaal mee oneens
- Deels mee oneens
- Geen mening
- Deels mee eens
- Helemaal mee eens

18. **Het is waarschijnlijk dat ik ziek word via plaagdieren in en om mijn woning**

*Markeer slechts één ovaal.*

- Helemaal mee oneens
- Deels mee oneens
- Geen mening
- Deels mee eens

- Helemaal mee eens

19. **Een ziekte opgelopen via plaagdieren in en om mijn woning leidt tot gezondheidsproblemen.**

*Markeer slechts één ovaal.*

- Helemaal mee oneens
- Deels mee oneens
- Geen mening
- Deels mee eens
- Helemaal mee eens

20. **De kans dat ik ziek word via plaagdieren in en om mijn woning is groot.**

*Markeer slechts één ovaal.*

- Helemaal mee oneens
- Deels mee oneens
- Geen mening
- Deels mee eens
- Helemaal mee eens

21. **Mijn zelfbeeld zou veranderen als ik ziek zou worden door plaagdieren in en om mijn woning**

*Markeer slechts één ovaal.*

- Helemaal mee oneens
- Deels mee oneens
- Geen mening
- Deels mee eens
- Helemaal mee eens

22. **Het oplopen van een ziekte via plaagdieren in en om mijn woning zou mijn leven sterk beïnvloeden**

*Markeer slechts één ovaal.*

- Helemaal mee oneens
- Deels mee oneens
- Geen mening
- Deels mee eens
- Helemaal mee eens

23. **Ik maak me zorgen dat plaagdieren in en om mijn woning mij ziek maken.**

*Markeer slechts één ovaal.*

- Helemaal mee oneens
- Deels mee oneens
- Geen mening
- Deels mee eens
- Helemaal mee eens

24. **De gedachte dat ik ziek kan worden door plaagdieren in en om mijn woning is beangstigend**
*Markeer slechts één ovaal.*

- Helemaal mee oneens
- Deels mee oneens
- Geen mening
- Deels mee eens
- Helemaal mee eens

## Plaagdierbestrijding

25. **Het bestrijden van plaagdieren voorkomt problemen in de toekomst**
*Markeer slechts één ovaal.*

- Helemaal mee oneens
- Deels mee oneens
- Geen mening
- Deels mee eens
- Helemaal mee eens

26. **Het is lastig voor mij om plaagdieren te bestrijden**
*Markeer slechts één ovaal.*

- Helemaal mee oneens
- Deels mee oneens
- Geen mening
- Deels mee eens
- Helemaal mee eens

27. **Het is gunstig voor mij om plaagdieren te bestrijden**
*Markeer slechts één ovaal.*

- Helemaal mee oneens
- Deels mee oneens
- Geen mening
- Deels mee eens
- Helemaal mee eens

28. **Ik weet hoe plaagdieren effectief bestreden kunnen worden**
*Markeer slechts één ovaal.*

- Helemaal mee oneens
- Deels mee oneens
- Geen mening
- Deels mee eens

- Helemaal mee eens

29. **Als ik plaagdieren in of om mijn woning heb, dan is het mijn bedoeling om die te bestrijden.**
*Markeer slechts één ovaal.*

- Helemaal mee oneens
- Deels mee oneens
- Geen mening
- Deels mee eens
- Helemaal mee eens

30. **Plaagdierbestrijding is niet de moeite waard.**
*Markeer slechts één ovaal.*

- Helemaal mee oneens
- Deels mee oneens
- Geen mening
- Deels mee eens
- Helemaal mee eens

31. **Het bestrijden van plaagdieren helpt in het beschermen tegen infectieziekten**
*Markeer slechts één ovaal.*

- Helemaal mee oneens
- Deels mee oneens
- Geen mening
- Deels mee eens
- Helemaal mee eens

32. **Plaagdierbestrijding kost mij te veel geld.**
*Markeer slechts één ovaal.*

- Helemaal mee oneens
- Deels mee oneens
- Geen mening
- Deels mee eens
- Helemaal mee eens

33. **Als ik plaagdieren in of om mijn woning heb, dan ben ik van plan die te bestrijden.**
*Markeer slechts één ovaal.*

- Helemaal mee oneens
- Deels mee oneens
- Geen mening
- Deels mee eens
- Helemaal mee eens

34. **Het bestrijden van plaagdieren in en om mijn woning kost veel tijd.**

*Markeer slechts één ovaal.*

- Helemaal mee oneens
- Deels mee oneens
- Geen mening
- Deels mee eens
- Helemaal mee eens

35. **Het is eenvoudig om plaagdieren te bestrijden**

*Markeer slechts één ovaal.*

- Helemaal mee oneens
- Deels mee oneens
- Geen mening
- Deels mee eens
- Helemaal mee eens

36. **Als ik plaagdieren in of om mijn woning heb, dan ga ik die bestrijden.**

*Markeer slechts één ovaal.*

- Helemaal mee oneens
- Deels mee oneens
- Geen mening
- Deels mee eens
- Helemaal mee eens

37. **Plaagdierbestrijding heeft vervelende consequenties**

*Markeer slechts één ovaal.*

- Helemaal mee oneens
- Deels mee oneens
- Geen mening
- Deels mee eens
- Helemaal mee eens

# Overlast plaagdieren

38. **Hoe zou u plaagdieren bestrijden?**

*Markeer slechts één ovaal.*

- Ik zou het zelf doen
- Ik vraag de gemeente om het te doen
- Ik huur een professioneel bedrijf in
- Niet bestrijden
- Anders: ____________________

39. **Voor het laatste deel van deze vragenlijst vragen we u aan te geven hoe vaak u in het afgelopen jaar de volgende plaagdieren in en om het huis heeft gehad?**

*Markeer slechts één ovaal per rij.*

| | Nooit | Een enkele keer | Een aantal keer | Regelmatig | Vaak |
|---|---|---|---|---|---|
| Knaagdieren (muizen, ratten e.d.) | | | | | |
| Vliegende insecten (muggen, vliegen, wespen e.d.) | | | | | |
| Kruipende insecten (kakkerlakken, vlooien, zilvervisjes e.d) | | | | | |
| Vogels (duiven en kraaiachtigen e.d.) | | | | | |
| Mollen | | | | | |

40. **Heeft u nog vragen en/of opmerkingen over deze vragenlijst?**

Dit was het einde van de vragenlijst, wij willen u hartelijk bedanken voor de medewerking!

## Einde vragenlijst

Mogelijk gemaakt door
Google Forms
