# Supplementary material for: Self-reported prevalence of pests in Dutch households and the use of the health belief model to explore householders’ intentions to engage in pest control
Source: PLoS One. 2017 Dec 28;12(12):e0190399. doi: 10.1371/journal.pone.0190399 (PMC5746277; doi:10.1371/journal.pone.0190399)
Supplement: S2 File — (PDF) [file pone.0190399.s002.pdf]

<u>Household pests questionnaire 2015</u>

Dear Participant,

Many people come into contact with pests in and around the home. Little is known about how often this occurs and how householders deal with the situation. That is why Utrecht University is studying the extent to which pests occur in Dutch households.

This short questionnaire is a first investigation. We would like to ask you a number of questions, completing the form will take 5 to 50 minutes. Your answers are ANONYMOUS and will be used only for this scientific study.

For the purpose of this survey the following animal and insects may be classed as pests:

- rodents (e.g. mice, rats etc.)
- flying insects (e.g. mosquitoes, flies, wasps, etc.)
- crawling insects (e.g. cockroaches, fleas, silverfish, etc.)
- birds (e.g. pigeons, crows, etc.)
- moles.

Thank you for your cooperation.

General questions

1. studentship

Are you presently in full time study? (Choose one answer)

Yes

No

2. area of residence

Where do you live? (Choose one answer)

Village

Town or city

Countryside

3. age

What is your age? ……

4. education

What is the highest level of education achieved? (Choose one answer)

Primary school

High school (3 options)

Vocational college

Higher vocational college

University

Other: …..

5. gender

What sex are you? (Choose one answer)

Male

Female

6. household pets

Do you keep animals? (Choose one answer)

No

Yes, I have one or more pet animals

Yes, I keep animals for a living

Yes I have both pets and animals that I keep for a living

7. (only for students) Living away from parental home

Do you live away from the parental home? (Choose one answer)

Yes

No

8. Year of construction

When was your house built? (Choose one answer)

Before 1960

After 1960

I don't know

9. Type of house

In which type of house do you live? (Choose one answer)

Terraced

Flat on 1st floor or higher

Ground floor flat

Semi-detached

Working farm

Detached house

Other: …..

Statements

Here are a number of statements that have to do with health, pests and pest control. Please indicate to what extent you agree with the statements by ticking one option. The answers are the same for each question, namely:

A completely agree
B partially agree
C neutral
D partly disagree
E completely disagree

Health statements

10. I follow my doctor’s instructions because they benefit my health

11. I eat a balanced diet

12. My health is important to me

13. I exercise regularly

14. I often do things to benefit my health (e.g. take exercise, go to bed early, eat a healthy diet)

15. Diseases that you can get from pests can be very serious

16. I think the chance of catching a disease from pests in or around my home is high

17. I expect to catch a disease from pests in and around my home within the next year.

18. It is likely that I become ill due to pests in and around my home

19. Catching a disease from pests in my home would lead to serious health problems

20. There is a good possibility of catching a disease from pests in my home

21. My self-esteem would be affected if I were to catch a disease from pests in my home

22. Catching a disease from pests in my home would influence my life greatly

23. I am concerned that pests in and around my home may make me ill

24. The thought that I could become ill from pests in and around my home is worrying

Pest control statements

25. Carrying out pest control prevents problems in the future

26. It is difficult for me to control pests

27. Exterminating pests is beneficial to me

28. I know how to control pests effectively

29. If I have pests in or around my home, I want to get rid of them

30. Pest control is not worth the effort

31. Pest control helps prevent the spread of disease

32. Pest control is too expensive for me

33. If I have pests in or around my home, I intend to use pest control

34. Pest control is time consuming

35. It is easy to control pests

36. If I have pests in my home, I will take action to control them

37. Pest control has unpleasant consequences

Questions on pests

38. How would you control pests in or around your home? (please tick one answer)

I would do it myself

I would ask the local authority

I would use a contractor

I would take no action

Other: …..

39. How often have the following pests been sighted in or around your home during the past year? (Please tick the appropriate box, once answer per pest category)

Pest category	never	once	a few times	regularly	often
Rodents (e.g. mice, rats etc.)					
Flying insects (e.g. mosquitoes, flies, wasps, etc.)					
Crawling insects (e.g. cockroaches, fleas, silverfish, etc.)					
Birds (e.g. pigeons, crows, etc.)					
Moles					

40. Do you have any questions or comments on this questionnaire?

This is the end of the questionnaire. Thank you very much for your participation.
