# Supplementary material for: Self-reported prevalence of pests in Dutch households and the use of the health belief model to explore householders’ intentions to engage in pest control
Source: PLoS One. 2017 Dec 28;12(12):e0190399. doi: 10.1371/journal.pone.0190399 (PMC5746277; doi:10.1371/journal.pone.0190399)
Supplement: S1 Table — (PDF) [file pone.0190399.s004.pdf]

S1 Table. Survey responses regarding pest prevalence.

		Frequency of prevalence in last year (%)				
Vermin Type	*Total*	**1** “Never”	**2** “Once”	**3** “A few times”	**4** “Regularly”	**5** “Often”
Rodents (mice, rats e.g.)	411	156 (37,8)	107 (25,9)	64 (15,5)	46 (11,1)	38 (9,2)
Flying insects (fly, wasps e.g.)	411	9 (2,2)	54 (13,1)	95 (23,0)	156 (37,8)	97 (23,5)
Crawling insects (roaches, silverfish e.g.)	407	60 (14,5)	118 (28,6)	95 (23,0)	76 (18,4)	58 (14,0)
Birds (pigeons, crow-likes e.g.)	407	173 (41,9)	74 (17,9)	36 (8,7)	75 (18,2)	49 (11,9)
Moles	409	332 (80,4)	37 (9,0)	11 (2,7)	18 (4,4)	11 (2,7)
