# Supplementary material for: Self-reported prevalence of pests in Dutch households and the use of the health belief model to explore householders’ intentions to engage in pest control
Source: PLoS One. 2017 Dec 28;12(12):e0190399. doi: 10.1371/journal.pone.0190399 (PMC5746277; doi:10.1371/journal.pone.0190399)
Supplement: S2 Table — (PDF) [file pone.0190399.s005.pdf]

S2 Table. Response frequencies to questions relating to the HBM.

		N (%)				
	**Mean**	**1**	**2**	**3**	**4**	**5**
**Health motivation (Crohnbach's alpha =.72)**						
1) I often do things to benefit my health	*4.1*	2 (0.5)	34 (8.2)	13 (3,1)	235 (56,9)	127 (30.8)
2) My health is important to me	*4.6*	11 (2.7)	5 (1.2)	1 (0.2)	107 (25.9)	288 (69.7)
3) I eat a balanced diet	*4.1*	8 (1.9)	34 (8.2)	15 (3.6)	216 (52.3)	137 (33.2)
4) I exercise regularly	*3.8*	35 (8.5)	66 (16.0)	16 (3.9)	142 (34.4)	152 (36.8)
5) I follow my doctor's instructions because they benefit my health	*3.9*	12 (2.9)	13 (3.1)	106 (25.7)	170 (41.2)	108 (26.2)
**Threat severity (Crohnbach's alpha =.61)**						
1) The thought that I could become ill from pests in and around my home is worrying	*2.3*	157 (38.0)	113 (27.4)	34 (8.2)	91 (22.0)	16 (3.9)
2) Catching a disease from pests in my home would influence my life greatly	*3.3*	37 (9.0)	73 (17.7)	92 (22.3)	153 (37.0)	53 (12.8)
3) My self-esteem would be affected if I were to catch a disease from pests in my home	*2.4*	142 (34.4)	77 (18.6)	90 (21.8)	81 (19.6)	17 (4.1)
4) Catching a disease through pests in my home would lead to serious health problems	*3.3*	59 (14.3)	44 (10.7)	90 (21.8)	154 (37.3)	65 (15.7)
5) Diseases that you can get from pests can be very serious	*3.6*	9 (2.2)	50 (12.1)	101 (24.5)	194 (47.0)	57 (13.8)
**Threat susceptibility (Crohnbach's alpha =.85)**						
1) I think the chance of catching a disease from pests in or around my home is high	*1.6*	246 (59.6)	116 (28.1)	29 (7.0)	18 (4.4)	2 (.5)
2) It is likely that I will catch a disease through pests in my house	*1.6*	237 (57.4)	115 (27.8)	31 (7.5)	25 (6.1)	1 (.2)
3) I am concerned that pests in and around my home may make me ill	*1.7*	248 (60.0)	99 (24.0)	22 (5.3)	37 (9.0)	4 (1.0
4) There is a good possibility that I will catch a disease through pests in my home	*1.5*	274 (66.3)	101 (24.5)	20 (4.8)	15 (3.6)	0 (0)
5) I expect to catch a disease from pests in and around my home within the next year	*1.2*	351 (85.0)	48 (11.6)	10 (2.4)	2 (.5)	0 (0)
**Perceived benefits (Crohnbach's alpha =.71)**						
1) I know how to control pests effectively [a]	*3.4*	5 (1.2)	44 (10.7)	19 (4.6)	221 (53.5)	48 (11.6)
2) It is easy to control pests	*3.7*	8 (1.9)	36 (8.7)	103 (24.9)	184 (44.6)	79 (19.1)
3) Exterminating pests is beneficial to me	*3.9*	10 (2.4)	31 (7.5)	46 (11.1)	214 (51.8)	107 (25.9)
4) Carrying out pest control prevents problems in the future	*3.9*	5 (1.2)	42 (10.2)	61 (14.8)	191 (46.2)	111 (26.9)
5) Pest control helps prevent the spread of disease	*4.1*	3 (.7)	47 (11.4)	39 (9.4)	172 (41.6)	151 (36.6)
**Perceived barriers (Crohnbach's alpha =.62)**						
1) Pest control is time consuming	*3.1*	56 (13.6)	91 (22.0)	55 (13.3)	164 (39.7)	45 (10.9)
2) It is difficult for me to control pests	*2.5*	98 (23.7)	113 (27.4)	108 (26.2)	75 (18.2)	17 (4.1)
3) Pest control is not worth the effort	*3.3*	25 (6.1)	74 (17.9)	100 (24.2)	186 (45.0)	25 (6.1)
4) Pest control is too expensive for me	*2.6*	69 (16.7)	158 (38.3)	76 (18.4)	97 (23.5)	11 (2.7)
5) Pest control has unpleasant consequences	*3.0*	49 (11.9)	102 (24.7)	95 (23.0)	141 (34.1)	24 (5.8)
**Intention questions (Crohnbach's alpha =.83)**						
1) If I have pests in my home, I want to get rid of them	*4.0*	5 (1.2)	44 (10.7)	19 (4.6)	221 (53.5)	118 (28.6)
2) If I have pests in my home, I intend to use pest control	*4.1*	3 (.7)	39 (9.4)	18 (4.4)	216 (52.3)	132 (32.0)
3) If I have pests in my home, I will take action to control them	*4.0*	7 (1.7)	50 (12.1)	16 (3.9)	222 (53.8)	116 (28.1)

Note: *Statements were scored as follows: 1) Absolutely disagree, 2) partly disagree, 3) No opinion, 4) Partly agree, 5) Absolutely agree.*

[a] Item deleted from subscale to improve internal consistency.
